# Efficacy of the transcutaneous electrostimulation in treatment dysfunctions of the TMJ associated with occlusion distortions

**DOI:** 10.1186/s12903-023-03662-z

**Published:** 2023-11-28

**Authors:** Zhanna Khachatryan, Tsovinar Hambartsoumian, Lyudmila Tatintsyan, Seda Burnazyan, Gagik Hakobyan

**Affiliations:** 1 Dept. of Therapeutic Stomatology, Yerevan State Medical University after M. Heratsi, Dental Clinic Pail, Yerevan, Armenia; 2 Dept. of Oral and Maxillofacial Surgery, Yerevan State Medical University after M. Heratsi, Yerevan, Armenia; 3 MC ArtMed, Yerevan, Armenia; 4 Dept. of Therapeutic Stomatology, Yerevan State Medical University after M. Heratsi, Yerevan, Armenia; 5 Dept. Oral and Maxillofacial Surgery, Yerevan State Medical University after M. Heratsi, 0028 Kievyan str. 10 ap. 65, Yerevan, Armenia

**Keywords:** Dysfunctions syndrome TMJ, Temporomandibular joint, Occlusion disorder

## Abstract

**Background:**

The study evaluation of the effectiveness the method of electrostimulation in treatment TMJ associated with occlusion disorders with the use of a patches by the company “Aganyan’’.

**Methods:**

The study included 54 patients with temporomandibular dysfunction syndrome who had previously undergone endodontic dental treatment. In patients temporomandibular disorders (TMD) determined on the basis of Diagnostic criteria for temporomandibular disorders(DC/TMD).All patients had occlusion disorders due to errors after dental filling restoration. To diagnose the TMJ, a CT scan was used. The complex therapy also included therapy and with the use of a patches by the company “Aganyan’’. The wearable patch includes a flexible substrate, a binder an adhesive layer, with an electrode foil attached to it. Patients applied one patch behind each TMJ. The patches were applied for eight hours every third day for three months. All the patients were given full-fledged endodontic treatment and restoration of the crown part, taking into account anatomical features.

**Results:**

The dynamics of the complex treatment of patients diagnosed with TMJ dysfunction syndrome showed that after treatment, the clinical symptoms gradually decreased and disappeared at the end of treatment. CT scan a year after treatment showed a normal ratio of TMJ elements. Сomplex treatment was effective in 87% of patients, after 3–5 months gradually decreased pain, noise in the joints, restriction of opening and closing of the mouth disappeared. Patients recovered their chewing functions, psycho-emotional state.

**Conclusion:**

The results of the studies revealed a positive effects for the complex treatment dysfunctions syndrome TMJ the using the patches by the company “Aganyan” through electrical stimulation with low intensity.

## Introduction

Temporomandibular joint (TMJ) disorders are a class of degenerative diseases of the musculoskeletal system connected with morphological and functional deformations. According to epidemiological data, TMJ disorders up to 25% of the population [1, 2].

TMJ disorders include anomalies in the intra-articular position and/or structure of the disc, as well as temporomandibular dysfunction (TMD) which is the second most common disease of the musculoskeletal system, causing pain and disability (after chronic low back pain) [3].

Disorders of the neuromuscular system causing pain are classified as masticatory pain dysfunction syndrome (MPDS) [4].

Clinical observations show that numerous factors may play role in the progression of TMD and degenerative changes connected with it. Ethiology comprex factors TMD include, trauma and micro trauma, rheumatoid arthritis, osteoarthritis, parafunction, bruxism, alocclusion, tooth loss occlusion **distortions** and functional overload, comorbid conditions [5–7].

Psychosocial factors include (anxiety, depression, and mental stress), socioeconomic status, and working conditions (including employment, occupation, working schedule, and working hours) sleep disorders and a busy lifestyle [8].

The influence of sleep bruxism with clinical manifestations of temporomandibular disorders (TMD) remains controver [9].

Clinical symptoms of dysfunctions syndrome TMJ is manifested may include one or more of the following symptoms: temporomandibular joint pain, restricted jaw movement and joint noise, otalgia, headache, neck pain and trismus, limited opening or deviation of the mandible upon opening and occlusal changes due to changes in the position of the mandible, [10] therefore, correct identification of symptoms and accurate diagnosis are essential for future treatment [8, 11].

Due to the complex and unique nature of each TMJ case, diagnosis requires an individual analysis of the patient, followed by various diagnostic modalities [12].

The purpose of the diagnostic protocol for TMD studies is to make a clinical diagnosis while simultaneously identifying other important characteristics that may influence presentation and treatment. The most widely used diagnostic protocol for TMD studies is the Diagnostic Criteria for Temporomandibular Disorders (RDC/TMD) based on the biopsychosocial model of pain [13, 14].

Similarly, it requires individual treatment taking into account specific characteristics of each patient’s disease.

Patient’s condition evaluation along with various imaging methods can help to determine the stage of disease, to diagnose and plan the treatment. The assessment of the TMJ begins with a thorough history and clinical examination. In some cases, clinical examination data is enough for the preliminary diagnosis and start conservative treatment. However, other patients will require diagnostic imaging of the TMJ to provide information that is not available on clinical examination [15].

As a result, TMJ identification can include any combination of the following diagnostic methods: panoramic radiography, Cone beam computed tomography (CBCT), Magnetic resonance imaging (MRI), Ultrasonography (US), arthrography [16–18].

CT scan is considered the most useful for visualizing the TMJ bones while MRI is considered the most useful for soft tissues visualization, including the disc and its articular ratio.

Arthrography is an invasive intra-articular examination; its usual indication is visualization of changes in the articular disc. Due to the inherent risk of this method, it has been superseded by MRI evaluation [19].

US examination, can be a useful option in the assessment of disc position in internal TMJ disorders. Ultrasound is indicated when MRI is contraindicated, to differentiate between TMJ and painful conditions of the major salivary glands [20, 21].

TMJ therapy is primarily aimed at eliminating pain, clicking joints, restoring TMJ functions. Research by various authors shows the relationship between temporomandibular disorders (TMD) and malocclusion [22].

Since in the etiology of dysfunction, a violation of occlusion is important to achieve the correct jaw relationship, it is necessary to restore the central ratio (CR). To stabilize the occlusion, occlusal splint therapy can used weth individual occlusal guards splint. The main goal of occlusal splint therapy is to separate the occlusion and relax the masticatory muscles. In this regard, occlusal correction has become a very popular method of choice for the treatment of or prevention TMJ [23]. Splinting therapy with occlusive splint not only reduces pain but also relieves the severity of symptoms [24].

When the compensatory capabilities of the masticatory and neuromuscular systems are overstrained, dysfunction occurs, leading to clinical symptoms and manifested by pain, strong clicking or limited mobility of the lower jaw, which forces the patient to seek help.

The polyethological nature of this pathology, the complexity of the clinical picture and the diagnosis and treatment connected with it, of course, should be complex: relief of emotional stress (psychotherapeutic treatment), medication, exercises of muscle groups, massage, pharmacotherapy and physiotherapy.

Myofascial pain is a common symptom and occurs in 31–76% of the population; it can be alleviated with massage, which relieves pain, relieves tension headaches and muscle pain, restores balance between masticatory muscle tension and improves chewing [25, 26]. Massage should be carried out twice a week, the duration of each session is at least 30 min.

Muscle training is a simple and non-invasive method of treating TMJ and should be done in moderation and the intensity should be increased over time to avoid pain and patient frustration with the proposed treatment.

Pharmacotherapy is considered to be a supportive therapy that complements other therapies to release the TMJ, reduce pain and inflammation in the joints and/or muscles.

Pharmacological agents are used for TMJ in the form anti-inflammatory drugs. The most commonly used drugs are muscle relaxants, nonsteroidal anti-inflammatory drugs (NSAIDs), analgesics, tricyclic antidepressants, benzodiazepines, and corticosteroids. Therefore, drugs, TMD such as NSAIDs and corticosteroids, which inhibit the release of inflammatory cytokines, are prescribed in TMD [27–31]. Glucocorticoids, usually diluted with local anesthetic, are most widely utilized for intra-articular injection to patients with TMJ for its potential advantages, such as its safe use, less systemic exposure, and few side effects [32].

Modern therapies use for treatments patients with dysfunctions of the TMJ a variety method, these procedures include neurotoxins, platelet-rich plasma, botulinum therapy, etc [33–36].

Among the also non-surgical methods used to treat TMJ physiotherapy, kinesiotaping, biofeedback [37–41].

A systematic review has shown that Low-level laser therapy (LLLT) is effective in relieving pain and improving functional outcomes in patients with TMJ [40, 41]. Popularity is the use of a LLLT that penetrates the skin with a wavelength of 904 nm and a frequency of 700 Hz to a depth of 30 mm.

LLLT exerts through multiple mechanisms of action, including facilitating the release of endogenous opioids, enhancing tissue repair and cellular respiration, increasing vasodilation and pain threshold, and reducing inflammation [42].

From non-invasive other methods of treatment, electrostimulation can be used for treatment methods in patients with dysfunctions of the TMJ and can improve the efficiency of treatment [43, 44].

Transcutaneous Electrical Nerve Stimulation (TENS) is a well-known method of pain relief for TMD. TENS is a method based on electrical stimulation of painful areas using surface electrodes and is considered safe and non-invasive. Due to the small number of studies (especially randomized), TENS cannot yet be considered a standard treatment for TMD, as its effectiveness has not yet been determined [45].

Electrical stimulation methods that exclude the use of large equipment and electrical wires or power supplies are relevant for the treatment of patients with TMJ disorders.

The research that we carried out was aimed at testing of the effectiveness the method of electrostimulation in treatment of dysfunctions of the TMJ with the use of a patches by the company “Aganyan”.

The potential difference causes electrons to flow across the surface of the skin from one of the electrodes to the other. In this sense, one of the electrodes can be considered as the cathode and the other electrode as the anode. The electrodes are made from various metals such as zinc and copper and are not connected to any electrical device such as a voltage or current source.

A potential difference can only be created when the electrodes are made of different metals. The main difference between Aganyan patches and existing methods of electrical stimulation is that these patches are not connected to any power source or batteries.

These ranges of voltages and electrical currents seem to be very safe for humans and do not have any side effects [46].

Purpose of the study evaluation of the effect using patches by the company “Aganyan” for treatment dysfunctions syndrome TMJ.

## Materials and methods

The study included 54 patients (males 28, females 26, the age range was 26–48 years with a mean SD of 35.2 years) with clinical features consistent with TMJ dysfunctions syndrome were enrolled who had previously undergone endodontic dental treatment. All patients presented occlusion disorders due to errors after dental filling restoration.

Patients examined for pain on palpation, at rest/during movement, muscle pain, sound on jaw movement, occlusion was also assessed. In patients temporomandibular disorders (TMD) determined on the basis of Diagnostic criteria for temporomandibular disorders(DC/TMD) [14].

### Inclusion criteria were

patients with the following symptoms, pain around the joint, in the pre-auricular region, in the muscles of mastication and the ear, limited or abnormal range of motion, and cranial and/or muscle pain, sound in the joint area while opening and closing the mouth.

Patients included in this study did not take any medications before starting treatment that could affect the results of the study.

### Exclusion criteria were

conditions after craniofacial trauma, rheumatic pathologies, surgery in the craniocervical region, neurological disease, uncontrolled systemic disease, any TMD treatment performed in the last 6 months, patients treated with corticosteroids or bisphosphonates.

Based on the clinical analysis of the patients with TMJ dysfunctions, can state that almost all the patients had occlusion disorders due to errors in dental filling. When examining the fillings, much attention was paid to the marginal fit of the filling, contact points, height in relation to the antagonist teeth, and the state of the periapical tissues. Based on x-ray studies, the quality of root canal filling was determined.

**Fig. 1:**
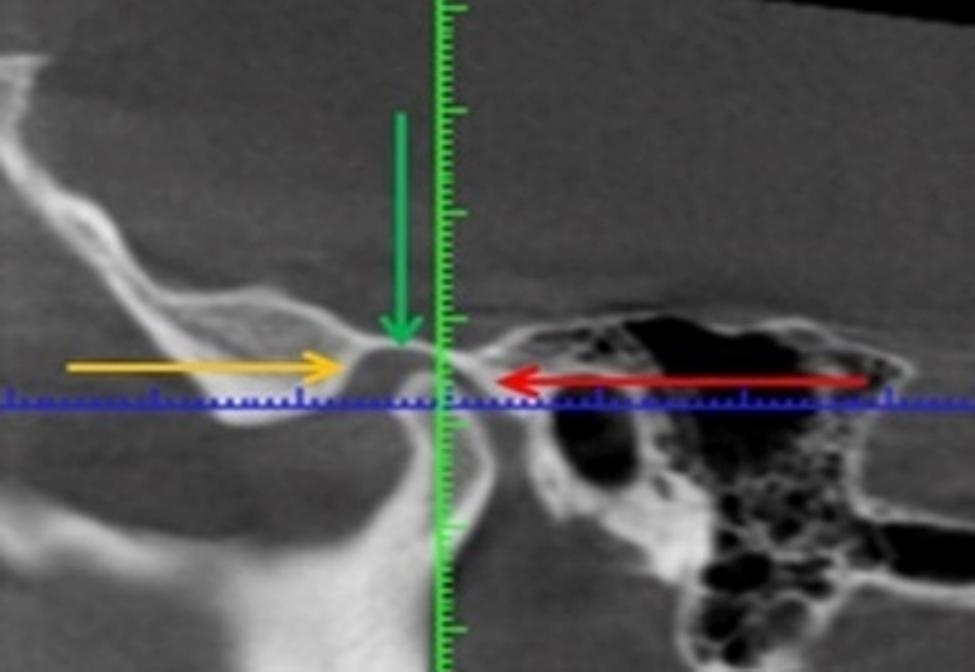
Computer tomography (CT) before treatment

To diagnose the TMJ a CBCT (Planmeca ProMax 3D Mid Helsinki, Finland) was used, which provided information about the condition of the tissues, the position of the axes of the joint heads (Fig. 1). CTBT scan diagnostics were performed by 3 doctors who were trained in viewing images of the TMJ; only one program was used to view CBCT scans.

Diagnostics of the TMJ condition was carried out by interpreting the obtained data. Normal function of the joints was characterized by noiseless movement of the head joint during rotation and progression. In this case, the disc is always on the joint head, moving with it. Contraction of the superior belly of the lateral pterygoid muscle causes an anteromedial effect on the disc, and the elastic resistance of the retrocondylar tissues causes smooth movement of the joint head and disc. Each patient’s course of treatment was worked out basing on the clinical and X-ray assessment of the disease, the causes preceding its development and occurrence. In any case, if the patient complained of pain in the TMJ area, both at rest and during the lower jaw movement, treatment began with its relief.

All the patients were given full-fledged endodontic treatment and restoration of the crown part, taking into account anatomical features under the control of X-ray diagnosis.

For endodontic treatment in patients used zinc oxide (ZnO), an antifungal drug (nystatin, fluconazole) and the probiotic “Narine” (Lactobacillus acidophilus n.v. as an antibacterial and antifungal agent in the composition of the paste. strain Er2 317/402), eugenol (clove oil), which was left in the root canal for 7 days, followed by filling with zinc-eugenol paste.To obtain the correct occlusal height, casts of the upper and lower jaws, diagnostic models were obtained, followed by their installation in the articulator, after the detection of supercontacts in the patient’s oral cavity, they were occlusal adjustments.

All the patients were treated symptomatically. The complex therapy included analgesics, non-steroidal anti-inflammatory drug, muscle relaxants and relaxation therapies, topical analgesics gel.

**Fig. 2:**
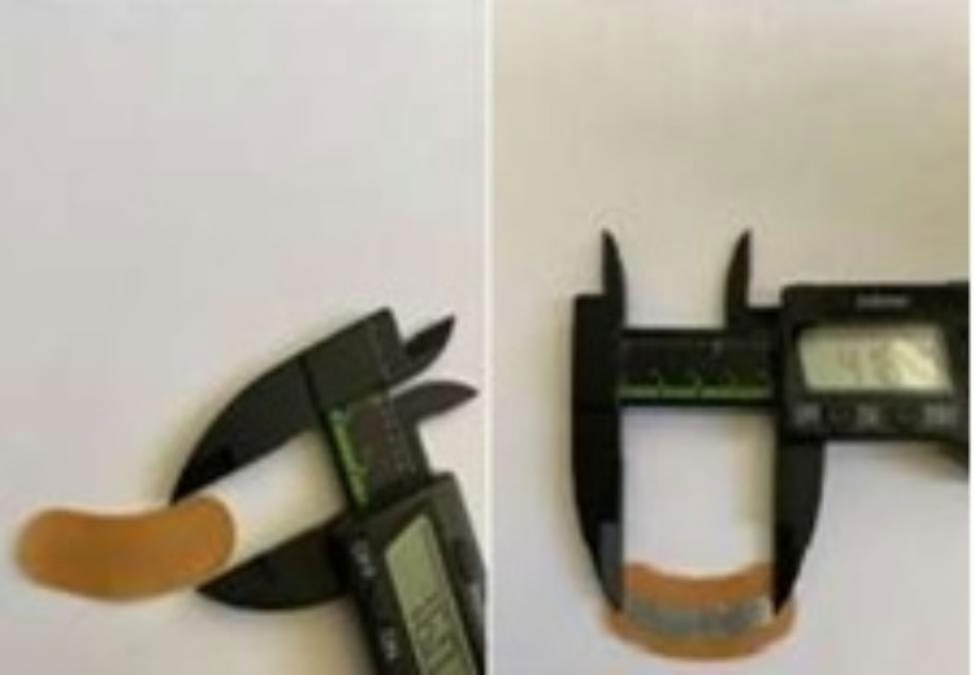
Patches by the company “Aganyan”

The complex therapy also included a patches by the company “Aganyan’’, patches includes a flexible substrate, a binder an adhesive layer, with an electrode foil attached to it (Fig. 2).

**Fig. 3:**
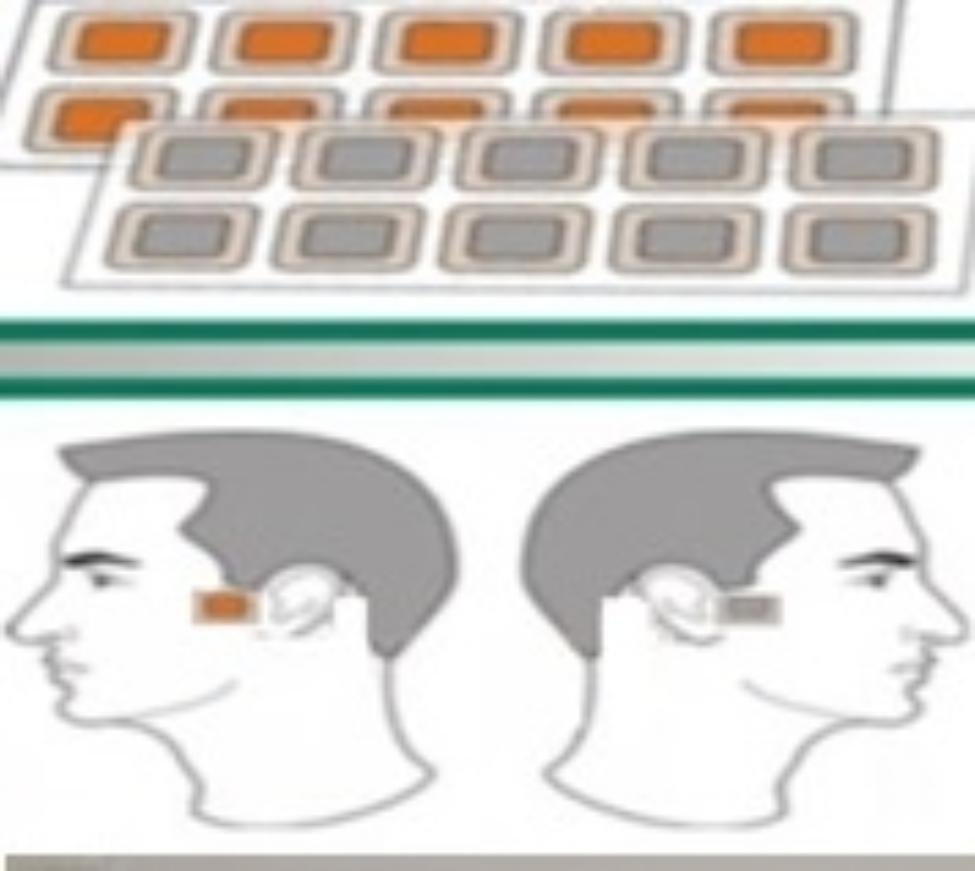
Scheme Patients applied 1 patch for each TMJ

The electrodes are made of zinc and copper. The potential difference causes electrons to flow across the skin surface from one of the electrodes to the other and create electrical currents. In this sense, one of the electrodes can be considered as a cathode, while another electrode can be considered an anode. These ranges of voltages and electrical currents appear to be very safe for humans. Patients applied 1 patch behind each TMJ 8 h every third day for three months (Fig. 3).

## Results

51 patients complained of pain around the joint, pre-auricular region, muscles of mastication and the ear region, while 37 patients complained of pain on opening the mouth during mastication, 24 patients had impaired movement of the jaws, mouth opening was normal in 43 patients and reduced in 9 patients, deviation of the mandible on closing was observed in 14 patients, 32 patients complained of sounds (Table 1).

**Table 1:** Clinical symptoms in the examined patients before treatment

Patient complaints	Pain on opening the mouth during mastication,	Impaired movement of the jaws	Mouth opening was normal	Mouth opening was reduced	Deviation of the mandible on closing	Complained of sounds
Total 51 patients	37	24	43	9	14	32

Complex treatment was effective in 87% of patients, after 3–5 months gradually decreased pain, noise in the joints, restriction of opening and closing of the mouth disappeared. Patients recovered their chewing functions, psycho-emotional state (Table 2).

**Table 2:** Clinical symptoms in the examined patients after complex treatment

Patient complaints	Pain on opening the mouth during mastication,	Impaired movement of the jaws	Mouth opening was normal	Mouth opening was reduced	Deviation of the mandible on closing	Complained of sounds
Total 51 patients	3	22	49	2	3	2

**Fig. 4:**
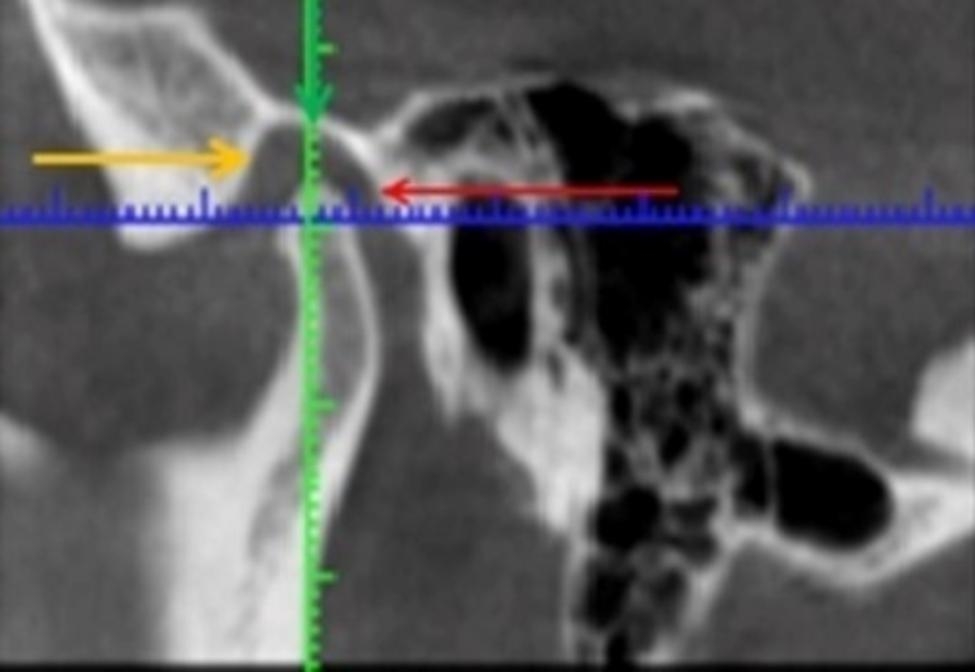
Computer tomography (CT) after 6 months treatment

CBCT scan after 6 months treatment showed a normal ratio of TMJ elements, the size of the bone parts of the joint and the joint space were normal (Fig. 4).

Data analysis showed a decrease in sound in the dynamics of treatment and normalization of parameters 3 months after treatment.

## Discussion

The increase in the number of patients with Temporomandibular dysfunction can be explained by the improvement of diagnostic and paraclinical methods, particularly due to technical medical research [49].

Temporomandibular dysfunction (TMJ) is a common disorder,most the leading symptoms is pain, followed by restricted mandibular movements, which can cause difficulty in eating or speaking; noises from the temporomandibular joints during jaw movement [50, 50].

The TMJ) disease is more common in patients aged 20–40 and women are four times more likely to suffer from this disorder.e specific characteristics of the disease [51].

The etiology of TMD is multifactorial, мalocclusion and occlusal interference are one of the factors in the development of temporomandibular disorders, so occlusal correction is included in the complex treatment of TMJ [52, 53].

Diagnosis and treatment of TMJ remains a challenge, and there is still no consensus on many aspects, the most widely accepted and standardized tool for assessment and classification of TMD is Diagnostic Criteria for Temporomandibular Disorders (DC/TMD) [14]. Diagnostic Criteria for Temporomandibular Disorders (DC/TMD) contains a structural Axis-I contains a prescribed physical examination protocol to make a specific physical diagnosis of TMD in relation to the joints and muscles) and a biopsychosocial component (Axis-II contains several tools to assess the psychological state of the patient) [14].

An important part of the diagnosis of TMJ is the differential diagnosis from those clinically significant but unusual conditions that require urgent treatment [54, 55].

Due to the complexity of the for many aspects etiology of TMD diagnosis, there is no consensus on treatment, depending on the type of TMJ a wide range of treatment methods are used: from conservative options to open surgical interventions, however, choosing the optimal one remains a problem.

From variety of conservative treatment options it has been shown that the use of occlusal splint reduces the intensity of pain and has a positive effect reduces the intensity of pain and has a positive effect [24].

For the treatment of patients in this category there are no uniform standards and treatment protocols, usually used, the non-surgical path is the first and main part. It consists of pharmacological therapies such as nonsteroidal anti-inflammatory drugs (NSAIDs), antidepressants and muscle relaxants and non-pharmacological treatment: biofeedback, LLLT, TENS [25–47].

It is well known that TENS can reduce pain in patients with TMD, but requires further study [58].

Mechanism of effect high frequency TENS is associated with a decrease in the amount of nociceptive substances released in peripheral tissues with the release of enkephalins and β-endorphins in the descending pain modulating system [59].

Since there are reports in the literature about the effectiveness of the method of electrical nerve stimulation for the treatment of chronic musculoskeletal pain, based on analogues of this technique, Aganyan patches have been included in complex therapy patients with TMJ dysfunction syndrome.

In this article, the authors, present the results of their own studies. The study included 54 patients with TMJ dysfunction syndrome who had previously undergone endodontic dental treatment. To evaluate the effectiveness of the treatment of patients with temporomandibular joint dysfunction, along with clinical and radiological methods was used. According to the analysis of clinical data and anamnesis, the main etiological factor TMJ dysfunction syndrome in the study group of patients was occlusal **distortions**. The main patient complaints included pain, difficulty or discomfort when biting or chewing, clicking, popping or grinding sound when opening or closing the mouth, ear pain, headache, decreased ability to open or close the mouth.

It is known that the violation of occlusion can significantly complicate the course of TMJ disease, and incorrect treatment tactics lead to instability of the formed occlusion and relapse of the disease. The pain is caused by abnormal contractions of the masticatory muscles, which can stimulate the production of inflammatory substances around the joint.

Complex treatment methods of the examined patients included correction of the occlusal height of filled teeth, the complex therapy also included therapy and with the use of a patches by the company “Aganyan’’,muscle relaxant, drug treatment, if indicated, orthopedic elimination of occlusal disorders.

Adequate psychotherapy, elimination of traumatic factors, the use of pharmacotherapeutic agents, normalization of functioning associated with occlusive factors, electrostimulation using of a patches by the company “Aganyan’’, made it possible to rehabilitate patients with impaired TMJ function.

The uniqueness of this invention is that electrical stimulation can be used for long-term stimulation TMJ with low voltage and current without the use of wires and batteries.

The strengths of this study mainly relate to the use of an alternative, side-effect-free technique to treat this category of patients using, limitations in this study are the lack of a control group with placebo.

It is important to note that this is the first report in the literature on the use of a patches by the company “Aganyan” in patients with TMJ and further research is needed to determine the effectiveness of the proposed technique and to make recommendations for its use.

When planning therapeutic measures for TMJ dysfunctions, it is necessary to carry out a clinical and radiological assessment, orthopantomography, CT scan, as well as eliminate the causes preceding its development (occlusal jaw ratios), which will significantly increase the possibility to diagnose and treat dysfunction of the temporomandibular joint.

## Conclusion

The results of the studies revealed a positive effects for the complex treatment dysfunctions syndrome TMJ the using the patches by the company “Aganyan” through electrical stimulation with low intensity.

## Data Availability

All data generated or analysed during this study are included in this published article.

## Abbreviations

DC/TMD: Diagnostic criteria for temporomandibular disorders
MPDS 0.4: Masticatory pain dysfunction syndrome
TMD: Temporomandibular dysfunction
MRI: Magnetic resonance imaging
CBCT: Cone beam computed tomography
MRI: Magnetic resonance imaging
US: Ultrasonography
CR: Central ratio
NSAIDs: Nonsteroidal anti-inflammatory drugs
LLLT: Low-level laser therapy
TENS: Transcutaneous Electrical Nerve Stimulation
ZnO: Zinc oxide
